# The underlying molecular mechanisms of Fyn in neonatal hypoxic-ischaemic encephalopathy

**DOI:** 10.3389/fncel.2024.1476856

**Published:** 2024-11-27

**Authors:** Jiao Zhou, Xiang Lu, Haichuan Wang

**Affiliations:** ^1^Department of Reproductive Medicine Nursing, West China Second University Hospital, Sichuan University, Chengdu, China; ^2^Key Laboratory of Birth Defects and Related Diseases of Women and Children (Sichuan University), Ministry of Education, Chengdu, China; ^3^Department of Cardiology, The First People’s Hospital of Yuexi County, Yuexi, China; ^4^Department of Paediatrics, Sichuan Academy of Medical Science and Sichuan Provincial People’s Hospital, School of Medicine, University of Electronic Science and Technology of China, Chengdu, China

**Keywords:** Fyn, HIE, molecular mechanism, pathogenesis, brain

## Abstract

Fyn is a cytoplasmic tyrosine kinase (TK) that is a nonreceptor and a member of the Src family of kinases (SFKs). It is involved in several transduction pathways in the central nervous system (CNS), such as oligodendrocyte development, myelination, axon guidance, and synaptic transmission. Owing to its wide range of activities in the molecular signaling pathways that underpin both neuropathologic and neurodevelopmental events, Fyn has remained of great interest for more than a century. Accumulating preclinical data have highlighted the potential role of Fyn in the pathophysiology of neonatal hypoxic-ischaemic encephalopathy (HIE). By mediating important signaling pathways, Fyn may control glutamate excitotoxicity, promote neuroinflammation and facilitate the death of neurons caused by oxidative stress. In this review, we address new evidence regarding the role of Fyn in the pathogenesis of this condition, with the aim of providing a reference for the development of new strategies to improve the prognosis of neonatal HIE. In addition, we also offer insights into additional Fyn-related molecular mechanisms involved in HIE pathology.

## Introduction

Neonatal hypoxic–ischaemic encephalopathy (HIE) is a relatively common cause of death and disability in newborns. The incidence of neonatal HIE in full-term infants ranges from 1 to 2 per 1,000 infants (Wang et al., 2019). In countries with a relatively high prevalence of poverty, the incidence of neonatal HIE ranges from 10 to 20 per 1,000 live births (Greco et al., 2020); moreover, 15–20% of the afflicted neonates pass away during the perinatal stage. Furthermore, 25% of patients have neurological disabilities as children, which places a substantial burden on families and society (Filippi et al., 2018; Gunn and Thoresen, 2019; Novak et al., 2019).

In preclinical studies, neonatal HIE animal models have been established in postnatal day (P) 7–10 rodents via unilateral common carotid artery ligation followed by systemic hypoxia. This leads to injury in the cortex, hippocampus and striatum ipsilateral to the ligation site (Rice et al., 1981; Sheldon et al., 1998). Recent studies have shown that hypoxic-ischaemic (HI) injury causes a rapid decrease in N-methyl-D-aspartate receptor (NR) 2A levels, resulting in NR2B tyrosine phosphorylation in the rat brain at P7. Following HI, NR2B expression decreases at P21, but NR2A and NR2B tyrosine phosphorylation increases (Gurd et al., 2002). Moreover, Src family kinases (SFKs) are activated in response to HI injury in neonatal mice. SFK activity is correlated with increased NR2A and NR2B tyrosine phosphorylation. Additionally, Fyn is an SFK that is expressed in neurons and glia in the nervous system (Bare et al., 1993), and Fyn levels are strongly correlated with increased NR2A and NR2B phosphorylation in response to HI injury. Although it is uncertain whether SFKs are responsible for these phosphorylation events, previous studies have shown that, in mice, NR2B and Fyn are expressed at much higher levels in both synaptic and extrasynaptic membranes at P7 than in the adult brain, suggesting the importance of Fyn in regulating NR2B expression in the developing brain (Jiang et al., 2011). To address the specific contribution of Fyn, Fyn was overexpressed in the neurons of neonatal mice subjected to HI-induced injury. Compared with control mice, Fyn-overexpressing mice presented severe brain damage and increased mortality following HI-induced injury. This was associated with increased tyrosine phosphorylation of NR2A and NR2B as well as increased calpain activity. In addition, NR2B phosphorylation at Y1252 and Y1472 was greater in the synaptic fractions of Fyn-overexpressing mice than in those of control mice (Knox et al., 2013). These studies are consistent with the findings in the adult ischaemia literature, namely, that Fyn forms a complex with the NR during HI-induced injury.

In summary, future research on the functional effects of Fyn-mediated cell death on neonatal HI-induced injury and the related mechanisms will hasten the development of therapies that specifically target critical signaling nodes that mediate brain injury without impairing normal brain development, given the critical role of Fyn in neurodevelopment. Here, we review recent findings, highlight how Fyn might be involved in HIE, and provide further detail on how Fyn might be regulated under these circumstances. We draw attention to the necessity of investigating further Fyn-related molecular processes to deepen our understanding of HIE aetiology.

## The pathogenesis of HIE

The primary mechanisms of HIE-induced injury to the neonatal brain include: (1) excitotoxicity, (2) oxidative stress, and (3) inflammation, which collectively contribute to neuronal cell death via either apoptosis or necrosis. The injury can be divided into four phases following a hypoxic-ischaemic (HI) insult just before, during, or immediately after labor (peripartum period): (1) the primary energy failure phase (0–6 h after), (2) the latent phase (6–12 h after the HI insult), (3) the secondary energy failure phase (12–72 h after the HI insult), if homeostasis is not effectively restored, and (4) the tertiary phase, where neuronal cell death may continue for days to months after the initial injury (Pedroza-García et al., 2022).

Specifically, the primary phase is characterized by primary energy failure during hypoxic-ischaemic events, leading to harmful reactions such as ATP-dependent pump blockage, lactic acidosis, intracellular accumulation of calcium ions, excitatory amino acid release, toxic oedema formation, and necrosis in the most sensitive areas of the brain (Nuñez et al., 2018). Prior to the secondary phase, there is an incubation period, the energy recovery phase of resuscitation (Yang et al., 2020). However, after 6–72 h, the brain’s energy depletion process reoccurs in areas with greater resistance, and maintenance of excitotoxicity, a large influx of ionic calcium, increased activation of neuronal NO synthase, oxidative stress and mitochondrial dysfunction lead to secondary energy failure and programmed neuronal death through activation of the caspase pathway. It is characterized by the deterioration of mitochondrial function and an acute inflammatory response, leading to apoptosis at this phase (Peeples and Genaro-Mattos, 2022). The tertiary phase involves a sustained inflammatory response and epigenetic changes. In this pathological process, oxidative stress can lead to direct central nervous system damage and activate a cascade of inflammatory responses that promote the development of the tertiary phase; persistent inflammation then exacerbates damage (Zhao et al., 2016; Nuñez et al., 2018).

## Structure and activation of Fyn kinase

The Fyn kinase is a 59-kDa protein that has 537 amino acids (Resh, 1998). The Fyn gene, which is located on chromosome 6q21, encodes the Fyn protein (Sherratt et al., 1997) and, as a member of the Src family, was initially identified in 1986 as Syn or Slk (Davidson et al., 1994). It is expressed mainly in the cytoplasmic leaflet of the plasma membrane, where it phosphorylates tyrosine residues on important targets involved in numerous signaling pathways (Saito et al., 2010). Three active isoforms of Fyn are known to exist: Fyn-B, Fyn-T, and Fyn-D7 (Goldsmith et al., 2002). Although Fyn-B is expressed throughout the body, the brain has a notably high concentration of Fyn-B (Goldsmith et al., 2002). Conversely, peripheral blood mononuclear cells and cells of hematopoietic origin are generally found to include Fyn-T and Fyn-D7, respectively (Meur and Karati, 2024). The catalytic domain (SHA1) of all Fyn isoforms is identical, but the linker sequences between the SH1 and SH2 domains are different in Fyn-B and Fyn-T, and unlike Fyn-B, Fyn-D7 does not have residues 233–287 (Meur and Karati, 2024). The primary cause of the structural organization differences between the canonical Fyn kinase and the Fyn-B, Fyn-T, and Fyn-D7 isoforms is alternative splicing. Exon 7A is added to Fyn-B, giving it a longer N-terminal unique domain; moreover, exon 7B is present in Fyn-T, whereas exon 7 is absent in Fyn-D7 (Goldsmith et al., 2002).

**Figure 1:**
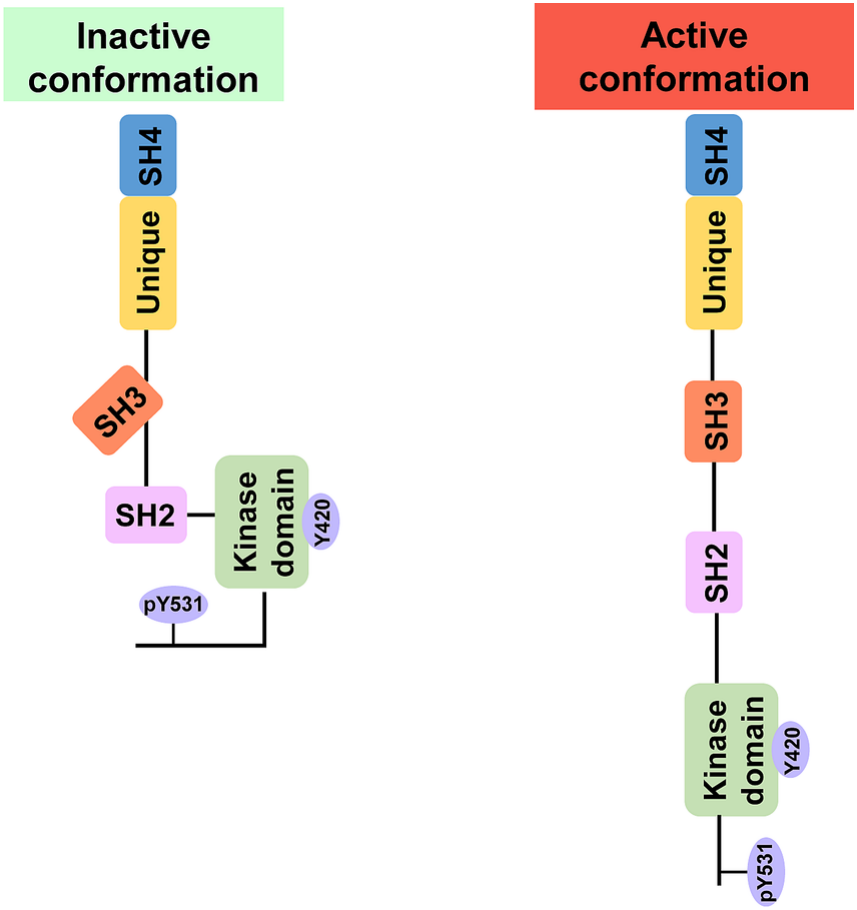
Inactive and active conformations of Fyn. Phosphorylation of Y531 in the C-terminus causes intramolecular contacts that inhibit kinase activity and protein–protein interactions, whereas phosphorylation of Y420 results in an open structure that is catalytically active and accessible to binding partners.

Fyn is composed of a unique domain that is distinct from Fyn and the four Src homology (SH) domains (SH1–SH4) that are highly conserved among SFK members (Resh, 1993). The capacity of Fyn to attach to the hydrophobic cellular membrane is made possible by the incredibly short SH4 domain at the N-terminus, which also controls the protein’s subcellular localization, stability, and activity through the process of palmitoylation (Resh, 1993; Zhang Y. et al., 2021). A unique domain consisting of the Tyr28 motif follows the SH4 domain. The Tyr28 motif is phosphorylated by the platelet-derived growth factor (PDGF) receptor, which activates Fyn (Yeo et al., 2011). The function of FYN is regulated by the dynamic phosphorylation of the Tyr531 motif at the C-terminus and the Tyr420 motif on the activation loop of the SH1 domain (Ponniah et al., 1999). When the Tyr531 motif is dephosphorylated, the intramolecular complex of Tyr531-SH2 disintegrates, leading to an open conformation that facilitates Fyn activation (Bhandari et al., 1998) (Figure 1). Because of its open conformation, Fyn can interact with a wide variety of substrates through the SH2 and SH3 domains, playing an important role as an upstream signaling mediator in numerous intracellular processes (Wu et al., 2007).

## Fyn in the brain

One of the most highly expressed SFKs in the brain, Fyn, is found in the striatum, cerebellum, and limbic regions of the brain (Umemori et al., 1992; Yagi et al., 1993b). Considerable amounts of Fyn have been found in the cerebral cortex, cerebellum, telencephalon, and brain stem throughout embryonic development (Yagi et al., 1994). This extensive Fyn distribution is evident during brain development and persists in the adult brain (Umemori et al., 1992). Fyn plays a crucial role in the brain, as evidenced by its widespread distribution. In fact, it modulates processes involved in learning and memory and interacts with excitatory and inhibitory synaptic transmission stimuli (Bixby and Jhabvala, 1993). Consistently, Fyn-deficient mice show an aberrant distribution of neocortical neurons at E16, specifically in layers II–III (Yuasa et al., 2004), with a diminished quantity of oligodendrocytes, axonal degeneration, and a thin cerebral cortex (Goto et al., 2008). Mice with a Fyn mutation in the regulatory residue Tyr531 (Y531F) exhibit a variety of abnormal locomotive behaviors, including hyperactivity leading to body weight loss, persistent tremors, lack of coordination, and early death (Yagi et al., 1993b). Furthermore, mice with the point mutation FynR176A in the SH2 domain exhibit poor neuronal migration in the cerebral cortex (Lu et al., 2015).

## Fyn function in cells and brain diseases

Fyn is implicated in cell growth, motility and adhesion; ion channel function; growth factor receptor signaling; platelet activation; and immune system regulation by modulating the proliferation and function of T cells, natural killer cells, bone marrow–derived macrophages (BMDMs), and mast cells (Schenone et al., 2011; Poli et al., 2018). For example, Fyn is also involved in T-cell differentiation, specifically proinflammatory cytokine release via Th17 cells (Ueda et al., 2012). In the central nervous system (CNS), Fyn participates in several processes related to brain development, including the differentiation of oligodendrocytes, myelination, neurite outgrowth, axon–glial signal transduction and synaptic plasticity (Nakamura et al., 2001; Schenone et al., 2011; Poli et al., 2018). For example, pharmaceutical upregulation of Fyn activity may neutralize the inhibitory effects of myelin debris on remyelination processes. Furthermore, this upregulation may be beneficial in several crucial aspects of oligodendrogenesis promotion, with increased Fyn activity required for the migration of OPCs to damaged sites (Wolf et al., 2001; Rajasekharan et al., 2010), the facilitation of actin dynamics permitting oligodendrocyte maturation and contact with axons (Liang et al., 2004), and the subsequent production of myelin proteins (Peckham et al., 2016). In addition, Fyn knockout mice display impaired long-term potentiation (LTP) and memory formation as well as ethanol sensitivity (Schenone et al., 2011). Fyn overexpression and dysregulation have been associated with various tumors, including melanomas, breast cancer, and gliomas (Tintori et al., 2015).

Currently, emerging evidence points toward its crucial role in neurodegenerative disorders, including Alzheimer’s disease (AD) and Parkinson’s disease (PD) (Guglietti et al., 2021). In both AD and PD, the current body of evidence promotes the inhibition of Fyn as a therapeutic target for pathological protein-induced toxicity and neuroinflammation. Specifically, inhibition of Fyn may interrupt Fyn-PrPc-ɑ-syn/Aβ signaling, perturbing protein aggregation and oligomeric binding, which would otherwise result in impaired neuronal communication and cell death (Kudo et al., 2012; You et al., 2012). Furthermore, given the key role that Fyn may play in the upregulation of microglial and astrocytic activation, Fyn kinase may represent a universal target, not only in AD and PD but also across multiple conditions known to be associated with a chronic neuroinflammatory response, including amyotrophic lateral sclerosis (ALS) (Liu and Wang, 2017), stroke (Zhang W. et al., 2021), traumatic brain injury (Schimmel et al., 2017) and status epilepticus (Sharma et al., 2021).

## Functional role of Fyn in the immature nervous system

Fyn has been extensively linked to neurodevelopment, and changes in the gene expression of Fyn have been connected to the emergence of deficiencies in the behavioral and emotional domains. Indeed, aberrant hippocampal development has been observed in mice lacking Fyn (Grant et al., 1992). Mice in which the Fyn gene is replaced also present increased sensitivity to stress and emotional abnormalities, which can be explained by the presence of lesions in the limbic system (Yagi et al., 1993a). Moreover, increased Fyn expression in the prefrontal cortex appears to be a consistent factor in the pathophysiology of schizophrenia (Ohnuma et al., 2003), and the Fyn gene sequence has been linked to numerous diseases that affect development (Schafer et al., 2019). In this respect, the Fyn polymorphisms rs6916861, rs3730353, and rs706895 are risk factors for schizophrenia in the Chinese–Han community (Wu et al., 2013), and rs6916861 and rs3730353 are risk factors for bipolar disorder (Szczepankiewicz et al., 2009). Additionally, Fyn regulates the NGF/TrkA and BDNF/TrkB pathways, both of which are implicated in neurodegenerative and neurodevelopmental processes (Peckham et al., 2016; Houlton et al., 2019). Notably, BDNF gene polymorphisms may contribute to the pathophysiology of autism spectrum disorder (ASD) via Fyn activity dysregulation (Iwasaki et al., 1998; Yoo et al., 2014). Fascinatingly, Fyn increases the phosphorylation of the semaphorin receptor (plexin) and facilitates the interaction between semaphorin and plexin (Renaud et al., 2008; Suda et al., 2011), which is crucial for mediating events related to neuronal polarization and migration (Renaud et al., 2008), synapse formation (Low et al., 2008; Orr et al., 2017), axonal pruning (Low et al., 2008) and dendritic arborization (Makihara et al., 2016; Ziak et al., 2020) during brain development.

## The implication of Fyn-related kinase pathways in HIE

### Neuroinflammation

Inflammation plays a crucial role in HIE and can mediate secondary neuronal death. The excessive activation of the inflammatory response in HIE is closely related to microglia, astrocytes, and neutrophils (Liu and McCullough, 2013). During ischaemia, neutrophils immediately infiltrate damaged brain tissue, exacerbating brain damage through various mechanisms, including the production of ROS, the release of proinflammatory cytokines, reduced microvascular flow due to neutrophil blockage of capillaries, and increased matrix metalloproteinase-9 (MMP-9) secretion, ultimately leading to breakdown of the blood–brain barrier (BBB) (Yao and Kuan, 2020; Bernis et al., 2023).

*In vitro* and *in vivo* evidence has revealed that Fyn can trigger microglial proinflammatory responses by regulating the PKCδ, MAPK, and NF-κB signaling pathways (Panicker et al., 2015). More precisely, Fyn in BV2 microglia can be quickly activated by LPS and TNF-α *in vitro*, which phosphorylate PKCδ at tyrosine 311 and activate it (Panicker et al., 2015). The Fyn-PKCδ pathway is activated in response to LPS and TNF-α, which phosphorylate the p38 and p44/42 (ERK) kinases. This, in turn, activates the NF-κB axis and causes the nuclear translocation of NF-κB p65 (Panicker et al., 2015), suggesting that Fyn is essential for proinflammatory microglial activity. Interestingly, as shown in Fyn knockout mice, Fyn is required for the release of proinflammatory cytokines, including IL-1β and TNF-α (Panicker et al., 2015). Treatment with 6-OHDA or MPTP results in decreased neuroinflammation, abnormal development of microglial and dopaminergic neuron contacts, and striatal dopaminergic nerve terminal degeneration in Fyn-or PKCδ-mutant animals. Fyn activation is inhibited by the use of TLR antagonists (IAXO-101) and TNF-α signaling antagonists (etanercept) (Panicker et al., 2015), suggesting that Fyn may be involved in TLR-and TNF-α-mediated pathways and that TLRs and TNF-α may be important upstream regulators of Fyn in HIE. Fyn expression is further increased by prolonged inflammatory stimulation, indicating the involvement of a positive feedback mechanism.

### Neuronal loss

Research has shown that mitochondrial dysfunction is the key to neurodegeneration in HIE (Li P. et al., 2018). Mitochondrial dysfunction leads to excessive accumulation of ROS, which can trigger the formation of the mitochondrial permeability transition pore (mPTP) (Lee et al., 2023; Zhou et al., 2023). The mPTP allows the release of proapoptotic factors, such as apoptotic protease activating factor-1 (Apaf-1) and cytochrome c, from the mitochondria into the cytoplasm. Cytochrome c interacts with Apaf-1 to form apoptotic bodies, activating cysteinyl aspartate-specific proteinase-9 (caspase-9) in the cytoplasm and initiating an apoptotic cascade, ultimately leading to apoptotic cell death (Hua et al., 2017; He et al., 2020).

One of the primary kinases linked to oxidative stress-induced neuronal death is the serine/threonine kinase PKCδ, which is cleaved by caspase-3 (Kaul et al., 2005). SFKs, such as Fyn, can increase the tyrosine phosphorylation of PKCδ, leading to its activation (Crosby and Poole, 2003). Interestingly, H_2_O_2_-induced proteolytic activation of PKCδ can be mediated by caspase-3 activation in dopaminergic neurons *in vitro*, and the general SFK inhibitor genistein was shown to inhibit H2O2-and MPTP-induced PKCδ tyrosine phosphorylation, proteolytic cleavage, and activation, thus protecting against dopaminergic neuronal apoptosis (Kaul et al., 2005). Furthermore, Fyn is rapidly activated in dopaminergic N27 rat cells exposed to dieldrin, an organochloride pesticide that has neurotoxic effects on dopaminergic neurons. This leads to PKCδ phosphorylation at tyrosine 311, which is essential for the catalytic activity of the enzyme. Furthermore, it triggers proteolytic cleavage and activation of caspase-3, which in turn promotes apoptosis in neurons (Saminathan et al., 2011).

Early attenuation of these processes by Fyn knockdown suggests that Fyn plays a critical role in dieldrin-induced apoptosis via PKCδ (Saminathan et al., 2011). *In vitro* activation of PKCδ in these studies demonstrated the proapoptotic involvement of Fyn in oxidative stress-induced neuronal degeneration. *In vivo* research and additional investigations using human cell lines, such as SH-SY5Y, are needed to fully understand the function of Fyn in HIE-induced neuronal death. Crucially, in animal models with Fyn deletion, these effects are accompanied by AMPK overactivation (Kang et al., 2017). Given that Fyn can inhibit AMPK activation via the PIKE and liver kinase B1 (LKB1) pathways (Yamada and Bastie, 2014), Fyn may promote excessive AMPK activation, which could lead to neuronal degeneration. The LKB1-AMPK-mTOR pathway has been shown to be essential for autophagy *in vitro* in SH-SY5Y cells treated with rotenone (Zhang et al., 2018), and Fyn could be considered an upstream regulator of this axis. Together, these findings lead us to hypothesize that, whereas Fyn overactivation may cause neuronal degeneration, Fyn activation may be necessary for dopaminergic neuronal survival. However, a study using a mouse model of 6-OHDA-induced Parkinson’s disease presented data suggesting that Fyn might not impact dopaminergic degeneration (Sanz-Blasco et al., 2018).

### Oxidative stress

ROS are produced mainly in mitochondria. Under normal circumstances, ROS are usually cleared by superoxide dismutase (SOD) and glutathione peroxide (GPX). During hypoxia, due to metabolic disruption, ROS cannot be immediately cleared by antioxidant enzymes, resulting in excessive accumulation of ROS (Greco et al., 2020).

In LPS-treated microglia, Fyn has been demonstrated to increase iNOS activation and nitrate release (Panicker et al., 2015), and the uptake of α-synuclein into microglia can cause the formation of ROS in the mitochondria (Panicker et al., 2015). Excessive ROS can be produced by impaired mitochondria due to inefficient mitochondrial respiration, and the activation of the NLRP3 inflammasome is significantly influenced by the formation of ROS within the mitochondria (Tschopp and Schroder, 2010). Fyn is an important factor in oxidative stress, as it links oxidative stress, neuroinflammation, and mitochondrial dysfunction together in the pathophysiology of HIE.

By phosphorylating Nrf2 at tyrosine 568, Fyn can increase its nuclear export and therefore suppress the expression of ARE-dependent genes (Niture et al., 2014). In this case, administering isorhynchophylline to SH-SY5Y cells treated with MPP^+^ decreased Fyn activation, which in turn inhibited the nuclear export of Nrf2, attenuating the neurotoxicity caused by MPP^+^ (Li Q. et al., 2018). In another study, therapy with isorhynchophylline also reduced the activation of glycogen synthase kinase (GSK)-3β (Li Q. et al., 2018), which is recognized as being upstream of Fyn. Specifically, GSK-3β can initiate the phosphorylation of Fyn, allowing it to enter the nucleus and phosphorylate Nrf2 directly, facilitating its nuclear export and destruction (Jain and Jaiswal, 2007). As previously noted, genistein prevents dopaminergic neuronal death *in vitro* by suppressing H_2_O_2_-induced PKCδ tyrosine phosphorylation, proteolytic cleavage, and activation (Kaul et al., 2005), further indicating the role of Fyn in the degradation of neurons caused by oxidative stress.

Furthermore, dieldrin, which exerts neurotoxic effects on the nigrostriatal pathway by inducing oxidative damage (Hatcher et al., 2007), increases dopaminergic neuronal death by quickly activating Fyn in dopaminergic neurons *in vitro*, which leads to PKCδ phosphorylation and subsequent caspase-3-mediated proteolytic cleavage and activation (Saminathan et al., 2011). It has been previously shown that, in LPS-treated BV2 cells, PKCδ is proteolytically cleaved by caspase-3 (Burguillos et al., 2011). Accordingly, oxidative stress-mediated dopaminergic cell death upon exposure to methylcyclopentadienyl manganese tricarbonyl requires proteolytic cleavage of PKCδ in a caspase-3-dependent manner (Anantharam et al., 2002).

### Glutamate excitotoxicity

Excitotoxicity is believed to be a common pathway for many neurological diseases and is associated mainly with the toxic effects of the excitatory neurotransmitter glutamate. Long-term activation of the glutamate receptor can trigger neurotoxicity, ultimately leading to loss of neuronal function and cell death (Neves et al., 2023).

In the context of HIE, pretreatment with the mGlu2/3R agonist LY354740 may reduce extracellular glutamate levels, prevent dopaminergic neuronal death, and prevent motor impairments in animals (Tan et al., 2020). Reduced NR2A and NR2B phosphorylation as well as Fyn downregulation followed these effects. Consequently, the opposite results were linked to the use of LY341495, an mGlu2/3R antagonist (upregulated Fyn, NR2A and NR2B phosphorylation; exacerbated motor impairment) (Tan et al., 2020). It has been demonstrated that mGlu3R activation increases glutamate uptake by encouraging the development of excitatory amino acid transporter 2 (EAAT2) in astrocytes (Yao et al., 2005). EAAT2 is essential for HIE-related excitotoxicity (Wei et al., 2019). Nevertheless, this process might be involved in the regulation of Fyn phosphorylation and NMDAR activation mediated by mGlu2/3R.

### Synaptic plasticity

The term “synaptic plasticity” describes how synapses, or connections between neurons, can change over time. Long-term potentiation (LTP), which involves repeatedly stimulating excitatory synapses to produce a persistent increase in synaptic strength, is an experimental model of synaptic plasticity (Malenka and Nicoll, 1999). When Fyn knockout (KO) mice are exposed to mild-intensity tetanus, hippocampal LTP is compromised (Grant et al., 1992). Remarkably, in Fyn-KO mice, LTP is normal up to the age of 14 weeks due to Src compensation, at which point compensatory Src expression is decreased and LTP deficit is apparent (Kojima et al., 1997). Mild stimulation can cause mice that overexpress constitutively active Fyn to exhibit a reduced threshold for LTP induction. According to these studies, Fyn modulates the threshold of LTP induction rather than being necessary for the initiation of LTP (Lu et al., 1999).

Fyn KO mice exhibit both LTP deficiency and poor spatial memory in the Morris water maze. Anatomically, Fyn deletion causes the dentate gyrus granule cells and CA3 target cells to be abnormally distributed (Grant et al., 1992). Additionally, after 3 months and 1 year of age, the hippocampal spine density is reduced in Fyn-KO mice (Babus et al., 2011). In adult Fyn KO mice, these structural modifications might be a factor in their abnormal hippocampal function.

### NMDA receptor surface expression and cleavage

Glutamate is the main excitatory neurotransmitter in the central nervous system (CNS) of mammals, and the NMDA receptor is a receptor for glutamate (Shen et al., 2022). High levels of glutamate in the synaptic gap bind to the NMDA receptor, and excessive excitation of the NMDA receptor leads to additional Ca^2+^ entering the cells, which causes the cells to become overexcited. The overexcitation of cells leads to the release of toxic substances, further promoting cell death and forming a vicious cycle. In addition, excessive stimulation of other glutamate receptor families can also trigger the influx of Na^+^ and Cl^−^, accompanied by the diffusion of water to counteract osmotic pressure, leading to cell swelling (Neves et al., 2023).

Typically, NRs are diheteromeric glutamate receptors composed of the modulatory subunits NR2A–D and the required NR1 subunit. NRs play a role in synaptic transmission, which is rapid and excitatory (Salter and Kalia, 2004), and generate substantial multiprotein assemblies at synaptic membranes (Husi et al., 2000). Multiple tyrosine residues on the C-terminal tails of the NR2A and NR2B subunits are phosphorylated by the SFKs Src and Fyn (Salter and Kalia, 2004). Moreover, NR currents are increased by exogenous Fyn, probably through tyrosine phosphorylation (Köhr and Seeburg, 1996). Kindling susceptibility and seizure occurrence are likewise regulated by Fyn-mediated NR tyrosine phosphorylation (Kojima et al., 1998). In addition, tyrosine phosphorylation of NR2B (pY NR2B) is decreased when Fyn is deleted, whereas pY NR2B is increased when Fyn is overexpressed. Interestingly, pY NR2A levels are normal in Fyn-KO mice but are elevated in Fyn-overexpressing mice (Kojima et al., 1998). These findings imply that Fyn may phosphorylate the NR2B subunit more favorably *in vivo*.

Fyn phosphorylates seven tyrosine residues in NR2B *in vitro* (Nakazawa et al., 2001). The expression of pY1070NR2B, pY1252NR2B, pY1336NR2B, and pY1472NR2B was shown to be highest in synaptic membranes in the developing brain, but pY1336NR2B was also detected in extrasynaptic membranes (Jiang et al., 2011). According to one study, synaptic lipid rafts contain more pY1252 NR2B than the postsynaptic density (PSD) (Delint-Ramirez et al., 2010). Although the physiological roles of pY1252 and pY1070NR2B are unknown, pY1336 stimulates NR2B calpain cleavage *in vitro* in response to glutamate exposure (Wu et al., 2007) and is linked to an increase in NR2B-phosphatidylinositol 3-kinase (PI-3 K) binding (Hisatsune et al., 1999).

Y472 is the most investigated Fyn-mediated NR2B tyrosine phosphorylation site. The phosphorylation of Y472 preserves NR2B-containing NR surface expression and synaptic localization because it is located inside the tyrosine endocytosis motif YEKL (Prybylowski et al., 2005; Nakazawa et al., 2006). Although pY1472 increases the surface expression of NR2B and decreases its endocytosis, it has little effect on excitatory synaptic transmission (Prybylowski et al., 2005; Nakazawa et al., 2006). Interestingly, mice with phenylalanine (Y472F) in place of Y472 exhibit normal LTP and spatial memory in the hippocampus but poor fear-related learning and diminished LTP in the amygdala (Nakazawa et al., 2006). Since Y472F animals exhibit 80% less tyrosine phosphorylation in the amygdala, pY472 impacts NR2B tyrosine phosphorylation (Nakazawa et al., 2006), with approximately 70% less phosphorylated tyrosine in the brain in Y472F mice than in WT mice (Knox et al., 2014). Additionally, the NR2B complex is altered in Y1472F mice, with reduced α-actinin and CaMKII linked to NR2B (Nakazawa et al., 2006). Finally, pY1472 causes the spinal cord and amygdala to activate the CaMKII pathway (Matsumura et al., 2010). Taken together, these findings imply that pY1472 influences complex formation, downstream signaling cascades, cell surface expression, and NR2B tyrosine phosphorylation.

## Conclusion

In conclusion, a growing body of convincing evidence indicates that Fyn plays a critical role in regulating the physiologic pathways and growth of neurons. These findings highlight the issues of whether and which changes to these molecular pathways cause neuronal deficits and dysfunctions following HIE, as well as whether and to what extent Fyn targeting can ameliorate these disorders. Research from numerous groups has provided answers to these questions, indicating that pharmacologically targeting Fyn may be therapeutically effective in treating brain damage where Fyn activity is compromised. Moreover, targeting established and new components of Fyn signaling cascades appears to be a promising therapeutic approach for preventing neuronal dysfunction following HIE. Given its widespread expression throughout the body and strong homology with other Src family kinases, which could result in unintentional off-target effects, Fyn is still a challenging factor to target.
